# 

**Published:** 1822-12

**Authors:** 


### MONTHLY CATALOGUE OF MEDICAL BOOKS

*** *It having been hinted to us that gentlemen, sending copies of their Works, prefer having their titles given at length, it is our intention, hereafter, to comply with this suggestion: but it is to be observed, that no books can be entered on this list except those sent to us for the purpose.*

*Since last month we have received the following books:*

Select Dissertations on several Subjects of Medical Science. By Sir Gilbert Blane, Bart. F.R.SS. Lond. Edinb. and Gotting.; Member of the Imp. Academy of Sciences of St. Petersburgh; and Physician to the King. Now first collected, with Alterations and Additions; together with several new and original Articles. —G. Underwood, London, 1822.

A Practical Essay on Diseases and Injuries of the Bladder; being that to which the Royal College of Surgeons adjudged the Jacksonian Prize for the Year 1821. Irritable Bladder is treated of in all its varieties, both with and without Mucous Discharges; also Inflammation, Suppuration, and Ulceration of that Organ; with Cancer and Stone, the Formation of which last is explained on entirely new Principles; Retention and Incontinence of Urine are considered very fully; and the whole is prefaced by an Inquiry on the mutual Influence that exists between Life and Organization, including some Observations on Ocular Spectra and the Nature of Mind. By ROBERT BINGHAM, Fellow of the Royal College of Surgeons, Author of Practical Essays on Strictures of the Urethra and Diseases of the Cutis, &c. &c. and Lecturer on the Theory and Practice of Surgery.—W. Simpkin and R. Marshall, and Burgess and Hill. 1822. pp. 467.

The Family Cyclopædia, or Manual of useful and necessary Knowledge, alphabetically arranged; comprising all recent Inventions, Discoveries, and Improvements, in Domestic Economy, Agriculture, and Chemistry; the most approved Methods of curing Diseases, with the Mode of Treatment in Cases of Drowning, other Accidents, and Poisons; Observations on Diet and Regimen; a comprehensive Account of the most striking Objects in Natural History, animate and inanimate; and a Detail of various Processes in the Arts and Manufactures; also a concise View of the Human Mind and the Passions, with their particular Application to our Improvement in Education and Morals. By JAMES JENNINGS. Second Edition, with Corrections and Additions.—Sherwood, Neely, and Jones, London, 1822. pp. 1376.

A Lecture, in which the Nature and Properties of Oxalic Acid are contrasted with those of Epsom Salts; or a very safe and effectual Method of preventing those fatal Consequences which have invariably followed the inadvertent Substitution of the former for the latter is submitted to the Public; together with the Symptoms, and the Treatment to be adopted when Oxalic Acid has been introduced into the Stomach, is fully investigated, and its Principles explained. By Robert Venables, M.B. of Trinity College, Dublin.—Callow and Wilson, 1822. pp. 56.

On the Use of Moxa as a Therapeutical Agent, by BARON D. I. LARREY, &c. &c. &c. Translated from the French, with Notes, and an Introduction containing a History of the Substance, by ROBLEY DUNGLISON, Fellow of the Royal College of Surgeons, &c. &c.—Underwood, 1822. pp. 148.

A new View of the Infection of Scarlet Fever, illustrated by Remarks on other Cutaneous Disorders. By WM. MACMICHAEL, M.D. F.R.S. Fellow of the College of Physicians, Physician Extraordinary to H. R. H. the Duke of York, and one of the Physicians of the Middlesex Hospital, London.—Underwood, 1822. pp. 100.

A Treatise on the Utility of Sangui-suction, or Leech-bleeding, in the Treatment of a great variety of Diseases, including the Opinions of eminent Practitioners, ancient and modern; with Instructions for the Process of Leeching; and an Appendix, delineating the characteristic Distinctions of true Leeches, with Directions for their Management and Preservation. By REES PRICE, M.D. Surgeon, Member of the Royal College of Surgeons, London, &c.—Simpkin and Marshall, 1822. p. 149.

---

### NOTICES TO CORRESPONDENTS.

*The Communications of* MR. MONEY, MR. JEFFREYS, MR. BAMPFIELD, MR. WARD, MR. MORLEY, MR. EDWARDS, MR. WALLER, MR. BUSH, *and* MR. EVANS, *have been received.*

*The Gentleman who refers to a paper in a former Number will perceive that we have given insertion to his Communication, having altered one word; the expression in his letter not being either literally in the paper referred to, nor, according to our judgment, is it implied. We request him to re-peruse the concluding observations.*

*The subject alluded to by our correspondent at Manchester is of great importance, as he very justly remarks, but he will find it noticed by various writers on Inflammation: at the same time, if he has any new light to throw upon the subject, we shall have much pleasure in laying it before the public.*

